# Cluster analysis application identifies muscle characteristics of importance for beef tenderness

**DOI:** 10.1186/1471-2091-13-29

**Published:** 2012-12-22

**Authors:** Sghaier Chriki, Graham E Gardner, Catherine Jurie, Brigitte Picard, Didier Micol, Jean-Paul Brun, Laurent Journaux, Jean-Francois Hocquette

**Affiliations:** 1 INRA, UMR1213, Recherches sur les Herbivores, Saint Genès Champanelle, F-63122, France; 2 INRA, VetAgro Sup, UMR1213, Recherches sur les Herbivores, Theix Saint Genès Champanelle, F-63122, France; 3 UNCEIA, Paris Cedex, 12,75595, France; 4 Beef CRC Murdoch University, Murdoch, WA, 6150, Australia

**Keywords:** Tenderness, Beef, Meta-analysis, Muscle biochemistry

## Abstract

**Background:**

An important controversy in the relationship between beef tenderness and muscle characteristics including biochemical traits exists among meat researchers. The aim of this study is to explain variability in meat tenderness using muscle characteristics and biochemical traits available in the Integrated and Functional Biology of Beef (BIF-Beef) database. The BIF-Beef data warehouse contains characteristic measurements from animal, muscle, carcass, and meat quality derived from numerous experiments. We created three classes for tenderness (high, medium, and low) based on trained taste panel tenderness scores of all meat samples consumed (4,366 observations from 40 different experiments). For each tenderness class, the corresponding means for the mechanical characteristics, muscle fibre type, collagen content, and biochemical traits which may influence tenderness of the muscles were calculated.

**Results:**

Our results indicated that lower shear force values were associated with more tender meat. In addition, muscles in the highest tenderness cluster had the lowest total and insoluble collagen contents, the highest mitochondrial enzyme activity (isocitrate dehydrogenase), the highest proportion of slow oxidative muscle fibres, the lowest proportion of fast-glycolytic muscle fibres, and the lowest average muscle fibre cross-sectional area. Results were confirmed by correlation analyses, and differences between muscle types in terms of biochemical characteristics and tenderness score were evidenced by Principal Component Analysis (PCA). When the cluster analysis was repeated using only muscle samples from *m. Longissimus thoracis* (LT), the results were similar; only contrasting previous results by maintaining a relatively constant fibre-type composition between all three tenderness classes.

**Conclusion:**

Our results show that increased meat tenderness is related to lower shear forces, lower insoluble collagen and total collagen content, lower cross-sectional area of fibres, and an overall fibre type composition displaying more oxidative fibres than glycolytic fibres.

## Background

In recent years, economic forces and competition from other animal proteins has put pressure on the beef industry to find ways of delivering a consistently high-quality product at the lowest cost. With respect to beef, tenderness has long been recognised as the key determinant of eating quality, with evidence demonstrating that consumers are willing to pay more for guaranteed tenderness [1].

Findings from the US National Beef Tenderness Survey [2] revealed a considerable variability in tenderness that depends, at least in part, on differences in muscle characteristics [3,4]. Indeed, this association between eating quality (i.e. tenderness) and muscle characteristics has arisen from observations that both variables vary between muscles in different species [5-7]. These differences between but also within animals are attributed to factors such as genetics, breed, sex, and muscle fibre type. Research so far has identified that muscle characteristics such as contractile fibre cross-sectional area, metabolic enzyme activity, collagen content, and solubility, as well as lipid content change as cattle mature, and also differ according to muscle types, feeding, exercise, breeds, and sexes [8-11]. Taking these observations into account, a collaborative group consisting of French scientists, French professionals, and European partners of the ProSafeBeef European programme (http://www.prosafebeef.eu/) have compiled all their data accumulated in the last 20 years from different experiments. This data warehouse, called BIF-Beef (Integrated and functional biology of beef), represents a new tool to explore phenotypic associations between animal growth, carcass composition, muscle tissue characteristics, and beef quality attributes of animals that are representative of French beef production [12]. However, we expect our results to be of a more general value and to apply outside the French data set.

Sensory analysis is generally considered as the reference method to evaluate eating quality. We assessed whether variability in beef tenderness (sensory analysis) could be explained by muscle fibre type, collagen characteristics, and other biochemical traits. Our hypothesis is that muscle fibre characteristics, collagen levels, and mitochondrial enzymatic activities do influence beef tenderness. However, controversies exist in the literature concerning the relationships between muscle fibres, connective tissue characteristics, and tenderness, depending on the experiment [3]. Consequently, in this meta-analysis, using the large volume of data available in the BIF-Beef database, we aimed to find a consistent relationship between these variables and tenderness across a range of muscles with focus on the *Longissimus thoracis* (LT) muscle.

## Methods

### Database description

The BIF-Beef data warehouse was initiated by researchers from the INRA (French National Institute for Agricultural Research) to create an internal database, named FiLiCol (for Fibres, Lipids, and Collagen), and contains data from numerous experiments where animal, carcass, muscle characteristics, and meat quality measurements were taken [13]. This database was compiled using data from other research programmes including QUALVIGENE (financially supported by APIS-GENE, a French private consortium) [14], and GEMQUAL (financially supported by the European Union) [15].

Currently, the BIF-Beef database contains about 331,745 measurements (including more than 15,764 measurements related to animal growth) of which 621 variables were observed across 5 muscle types from 5,197 animals (1–120 months of age) belonging to 20 different breeds, and from 43 different experiments. BIF-Beef has been described in detail in previous papers [12,16] and new data is continuously being added.

### Sensory analysis

In this study, the BIF-Beef data-set was clustered (or classified) into 3 tenderness groups (high, medium, and low) on the basis of trained taste panel tenderness scores of all meat samples consumed (4,366 observations from 40 different experiments). In experiments considered in this study, 14-day aged samples were grilled (55-60°C) and then tasted by trained panellists who rated them on non-structured line scales marked at the extremities ‘low’ and ‘high’ and subsequently scored as the distance in units of 1, from 0 to 10 [17-20].

### Studied muscles and breeds

Samples came from mainly French breeds (Table 1) including Aubrac, Salers, Limousin, Charolais × Salers, Charolais, Holstein, and Blond d’Aquitaine, and from different muscles (Table 1) including *Semitendinosus* (ST), *Semimembranus* (SM), *Rectus abdomin*is (RA), *Triceps brachii* (TB), and principally *Longissimus thoracis* (LT). These muscles are known to differ in the proportions of their muscle fibre types [21], collagen levels, and palatability [22].

**Table 1:** Number of measurements for different variables in seven studied breeds

**Low Tenderness**								
**Breed**	*Aubrac*	*Salers*	*Limousin*	*Charolais × Salers*	*Charolais*	*Holstein*	*Blond d'Aquitaine*	
**Variables**								**Total**
Tenderness scores	53	91	321	12	312	15	215	1019
WBSF^1^			281		141		197	619
Total Collagen	49	80	36		138	15	17	335
Insoluble Collagen	49	80	36		138	15	17	335
ICDH^2^	53	81	39	12	165	15	17	382
LDH^3^	53	81	39	12	165	15	17	382
FG (%)^4^	53	81	39	8	65	15		261
SO (%)^5^	53	81	39	8	65	15		261
CSA^6^	51	81	320	8	217	15	211	903
**Total**	414	656	1150	63	1601	120	725	
**Medium Tenderness**								
**Breed**	*Aubrac*	*Salers*	*Limousin*	*Charolais × Salers*	*Charolais*	*Holstein*	*Blond d'Aquitaine*	
**Variables**								**Total**
Tenderness scores	52	83	756	24	730	13	420	2078
WBSF^1^			697		535		413	1645
Total Collagen	51	82	58		38	13	4	246
Insoluble Collagen	51	82	58		38	13	4	246
ICDH^2^	52	82	59	16	146	13	4	372
LDH^3^	52	82	59	16	146	13	4	372
FG (%)^4^	52	82	59	2	59	13		267
SO (%)^5^	52	82	59	2	59	13		267
CSA^6^	52	82	750	2	595	13	418	1912
**Total**	414	657	2555	89	2514	104	1275	
**High Tenderness**								
**Breed**	*Aubrac*	*Salers*	*Limousin*	*Charolais × Salers*	*Charolais*	*Holstein*	*Blond d'Aquitaine*	
**Variables**								**Total**
Tenderness scores	21	66	286	12	525	2	357	1269
WBSF^1^			265		435		354	1054
Total Collagen	21	65	21		25	2		134
Insoluble Collagen	21	65	21		25	2		134
ICDH^2^	21	64	21	8	64	2		180
LDH^3^	21	64	21	8	64	2		180
FG (%)^4^	21	65	21	2	21	2		132
SO (%)^5^	21	65	21	2	21	2		132
CSA^6^	21	65	286	3	456	2	353	1186
**Total**	168	519	963	41	1729	16	1064	

### Biochemical and mechanical muscle traits

Within the muscles sampled, a range of different muscle fibre types, collagen to mechanical characteristics, and biochemical traits were reported. These included Warner-Bratzler Shear force (WBSF), activities of the metabolic enzymes lactate dehydrogenase (LDH) (representative of glycolytic metabolism), and isocitrate dehydrogenase (ICDH) (representative of oxidative metabolism), proportions of fast glycolytic (FG), and slow oxidative fibres (SO), mean cross-sectional area (CSA) of fibres, and lastly, total and insoluble collagen content (Tables 1 and 2).

**Table 2:** Number of measurements for different variables in five muscle types

**Low Tenderness**						
**Muscle**	LT^7^	ST^8^	TB^9^	RA^10^	SM^11^	
**Variables**						**Total**
Tenderness scores	778	123	87	15	16	1019
WBSF^1^	619	-	-	-	-	619
Total Collagen	136	106	74	3	16	335
Insoluble Collagen	136	106	74	3	16	335
ICDH^2^	176	103	76	11	16	382
LDH^3^	176	103	76	11	16	382
FG (%)^4^	141	123	87	15	16	261
SO (%)^5^	141	123	87	15	16	261
CSA^6^	690	110	76	11	16	903
**Medium Tenderness**						
**Muscle**	LT^7^	ST^8^	TB^9^	RA^10^	SM^11^	
**Variables**						**Total**
Tenderness scores	1839	82	85	65	7	2078
WBSF^1^	1645	-	-	-	-	1645
Total Collagen	132	75	72	10	7	296
Insoluble Collagen	132	75	72	10	7	296
ICDH^2^	149	82	81	53	7	372
LDH^3^	149	82	81	53	7	372
FG (%)^4^	103	74	73	10	7	267
SO (%)^5^	103	74	73	10	7	267
CSA^6^	1748	74	73	10	7	1912
**High Tenderness**						
**Muscle**	LT^7^	ST^8^	TB^9^	RA^10^	SM^11^	
**Variables**						**Total**
Tenderness scores	1157	41	24	46	1	1269
WBSF^1^	1054	-	-	-	-	1054
Total Collagen	62	41	21	9	1	134
Insoluble Collagen	62	41	21	9	1	134
ICDH^2^	76	40	23	40	1	180
LDH^3^	76	40	23	40	1	180
FG (%)^4^	59	41	21	10	1	132
SO (%)^5^	59	41	21	10	1	132
CSA^6^	1112	41	21	11	1	1186

Warner-Bratzler shear force was measured on cooked (55-60°C) meat after 14 days of ageing post-mortem [7,17].

The metabolic enzyme activities of muscles studied were determined by enzymatic activity of ICDH and LDH (μmole/min/g muscle). Enzyme activities were measured spectrophotometrically in all muscles studied, using the methods described by Piot *et al.*[23], Listrat *et al.*[24] and Jurie *et al.*[11]. Moreover, the proportions (% of SO and FG) and cross-sectional area (μm^2^) of muscle fibres were determined by histochemical methods [25,26].

Total and insoluble collagen content (mg/g dry matter) was determined using the method of Listrat *et al.*[24], described in detail by Listrat & Hocquette [27].

### Statistical analysis

To assess the relationship between tenderness and other muscle mechanical and biochemical traits, a cluster analysis was performed with all available data from five different muscles. The taste panel tenderness scores were initially clustered (FASTCLUS procedure in SAS, [28]) into three discrete classes corresponding to high (> 6.5), medium (> 5.2 and < 6.5), or low scores (< 5.2). These clusters were then used as a fixed effect in general linear models (GLM procedure in SAS) describing the other muscle mechanical and biochemical traits. In this manuscript, mean values (for WBSF, total and insoluble collagen contents, ICDH, LDH, proportions of FG and SO fibre types and CSA) are indicated after sorting data from each variable into clusters for tenderness, taking into account differences between numbers of data entries for each variable.

To ensure that these results were not being unduly biased by samples from tough muscles which always clustered in the lowest tenderness cluster, the analysis was repeated with data only from LT muscle.

In addition, a Principal Component Analysis (PCA) was performed with 495 samples from different clusters and from different muscle types for which data from all significant variables was present (namely tenderness score, total and insoluble contents, LDH and ICDH activities, proportions of the different muscle fibre types and CSA). This statistical method calculates new variables, called principal components, which are linear combinations of the original variables to account for the variability in the data based on the study of the covariances and the correlations between original variables [29]. Correlations between original variables were declared significant with correlation coefficients higher than 0.1218 (P < 0.05). Results are presented in a 2D projection graph where variables near each other at the periphery of the circle are positively correlated, and variables separated by 180° are negatively correlated. The closer the variables are to the periphery of the circle, the higher the coefficient of correlation between variables. Individuals are also presented in the same 2D projection. When some muscle samples are located in the same part of the projection as some variables, values of these samples for the considered variables are high.

## Results and discussion

In the current study, statistical analysis allowed us to discriminate 3 sensory tenderness clusters which were classified as low, medium, and high, and contained, respectively, 1,019, 2,078 and 1,269 samples of meat with tenderness values (Table 3). These clusters contained similar proportions of samples from the Limousin breed (23-36%), the Charolais breed (31-41%) and the Blonde d’Aquitaine breed (20-28%) (Table 1). Moreover, in each cluster there were mainly samples from the LT muscle with tenderness values of 76%, 88%, and 91% for the low, medium, and high tenderness classes, respectively (Table 2).

**Table 3:** Numbers (N), means and standard errors (SE) of three tenderness groups determined by the FASTCLUS procedure of SAS and the corresponding WBSF, collagen, muscle fibre, and biochemical traits in all five muscles combined (upper row) and in LT muscle only (lower row)

	**Low**	**Medium**	**High**
**Tenderness** (0–10 Scale)	**4.6**^**c**^± 0.6* (N=1019)	**5.9**^**b**^**± 0.4** (N=2078)	**7.1**^**a**^**± 0.5** (N=1269)
	**4.7**^**c**^**± 0.5** (N=871)	**6.1**^**b**^**± 0.3** (N=1749)	**7.1**^**a**^**± 0.5** (N=1154)
**WBSF**^1^ (N/cm^2^)	**46.1**^**a**^**± 1.6** (N=619)	**40.1**^**b**^**± 1.3** (N=1645)	**35.9**^**c**^**± 1.1** (N=1054)
	**45.7**^**a**^**± 0.3** (N=619)	**40.0**^**b**^**± 0.2** (N=1645)	**36.0**^**c**^**± 0.3** (N=1054)
**Total collagen** (mg/g dry matter)	**29.0**^**a**^**± 1.4** (N=335)	**27.6**^**b**^**± 1.5** (N=296)	**27.7**^**b**^**± 1.9** (N=134)
	**25.5**^**a**^**± 1.4** (N=136)	**23.7**^**b**^**± 1.3** (N=132)	**20.5**^**c**^**± 1.1** (N=62)
**Insoluble collagen** (mg/g dry matter)	**22.6**^**a**^**± 1.3** (N=335)	**22.5**^**a**^**± 1.2** (N=296)	**20.7**^**b**^**± 1.2** (N=134)
	**19.0**^**a**^**± 1.1** (N=136)	**19.0**^**a**^**± 1.1** (N=132)	**17.0**^**b**^**± 0.7** (N=62)
**ICDH**^2^ (μmole/min per g muscle)	**1.4**^**b**^**± 0.03** (N=382)	**1.6**^**a**^**± 0.03** (N=372)	**1.6**^**a**^**± 0.04** (N=180)
	**1.5**^**b**^**± 0.04** (N=176)	**1.7**^**a**^**± 0.04** (N=149)	**1.75**^**a**^**± 0.06** (N=76)
**LDH**^3^ (μmole/min per g muscle)	**938**^**a**^**± 10** (N=382)	**941**^**a**^**± 10** (N=372)	**941**^**a**^**± 14** (N=180)
	**978**^**a**^**± 15** (N=176)	**957**^**a**^**± 16** (N=149)	**940**^**a**^**± 22** (N=76)
**FG**^4^ (%)	**54**^**a**^**± 1.5** (N=261)	**53**^**ab**^**± 1.6** (N=267)	**52**^**b**^**± 1.3** (N=132)
	**52**^**a**^**± 1.2** (N=141)	**50**^**a**^**± 1.1** (N=103)	**50**^**a**^**± 1.3** (N=59)
**SO**^5^ (%)	**23**^**b**^**± 2.5** (N=261)	**25**^**a**^**± 2.4** (N=267)	**25**^**a**^**± 2.4** (N=132)
	**33**^**a**^**± 1.1** (N=141)	**33**^**a**^**± 0.9** (N=103)	**33**^**a**^**± 1.2** (N=59)
**CSA**^6^ (μm^2^)	**3336**^**a**^**± 18** (N=903)	**3057**^**b**^**± 15** (N=1912)	**2903**^**c**^**± 16** (N=1186)
	**3070**^**a**^**± 12** (N=690)	**2960**^**b**^**± 13** (N=1748)	**2814**^**c**^**± 13** (N=1112)

Differences between means were determined by ANOVA.

* Mean **±** SE.

^1^WBSF: Warner-Bratzler Shear Force; ^2^ICDH: isocitrate dehydrogenase; ^3^LDH: lactate dehydrogenase; ^4^FG: proportion of fast glycolytic muscle fibres; ^5^SO: proportion of slow oxidative muscle fibres; ^6^CSA: cross-sectional area of muscle fibres.

a, b, c : P < 0.05 as determined by ANOVA.

With the second cluster analysis (only data from LT muscle), low, medium, and high tenderness clusters contained, respectively, 871, 1,749 and 1,154 samples (Table 3). In this case, there was no difference in breed proportion but we had mostly young bulls (92% compared to 85% for the first analysis) in each cluster compared with the first analysis within all five muscle types. This was because samples from the QUALVIGENE experiment were represented by only LT muscle and were only taken from young bulls. Therefore, proportions of young bulls in each cluster were modified after excluding data from the four other muscles in the second analysis.

Finally, PCA performed with 495 samples from different clusters and different muscle types allowed us to analyse more precisely the correlations between tenderness score, total and insoluble contents, LDH and ICDH activities, proportions of the different muscle fibre types and CSA, as well as the distribution of samples according to the values of these key variables.

### Warner-Bratzler Shear force values

As expected, lower WBSF values were associated with more tender meat, and higher WBSF values were associated with less tender meat, with this effect evident in the analysis containing all muscle types as well as that of the LT muscle only (Table 3). These results align well with previous work [22,30-38] where the negative correlation between consumer tenderness and WBSF was clearly demonstrated despite a high variability in the correlation coefficient (−0.26 < r < −0.95).

### Connective tissue

Collagen is the major component of muscle connective tissue, and its association with meat tenderness has been the target of numerous studies [39,40]. In this study, muscles in the lowest tenderness class had the highest total collagen content (29.0 mg/g dry matter) with no differences in total collagen content between the medium and high classes (Table 3). Moreover, muscles in the highest tenderness group had the lowest insoluble collagen content (20.7 mg/g dry matter) and thus the highest soluble collagen content. However, there was no difference in insoluble collagen content between the medium and low tenderness classes (Table 3). Similar results were observed from data using the LT muscle only (Table 3), noting a significant difference in total collagen content amongst all three tenderness classes with respect to the LT.

**Figure 1:**
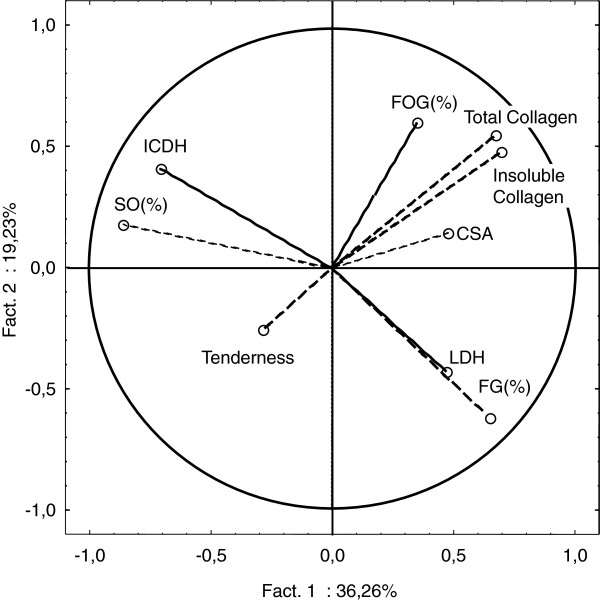
**Plot of the first two principal component score vectors showing relationships between muscular characteristics and tenderness with 495 samples from different clusters and muscle types.** Tenderness: tenderness score of grilled samples (55-60°C) after 14 days of ageing; Total collagen: total collagen content in mg/g dry matter; Insoluble collagen: insoluble collagen content in mg/g dry matter; ICDH: isocitrate dehydrogenase activity in μmole/min per g muscle; LDH: lactate dehydrogenase activity in μmole/min per g muscle; FG (%): proportion of fast glycolytic muscle fibres; FOG (%):proportion of fast oxydo-glycolytic muscle fibres; SO (%):proportion of slow oxidative muscle fibres; CSA: mean cross-sectional area of muscle fibres in μm^2^.

Correlation analysis indicated a strong positive correlation between total and insoluble collagen contents (r = +0.81), as expected, both variables being negatively correlated with tenderness score but with a moderate coefficient (r = −0.15 to -0.20, P < 0.05, Table 4 and Figure 1). On average, ST and TB muscles contained more total and insoluble collagen contents than LT, based on the distribution of samples on the plot of the first two principal component score vectors. Total and insoluble collagen contents were 2.84 and 2.43 mg/g dry matter, respectively, in LT, compared to 4.25 and 3.40 mg/g dry matter, respectively, in TB and 4.74 and 3.74 mg/g dry matter, respectively, in ST (P < 0.01 between the three muscles).

**Table 4:** Coefficient of correlations between the most significant variables

	**Tenderness**	**Total collagen**	**Insoluble collagen**	**ICDH activity**	**LDH activity**	**SO (%)**	**FOG (%)**	**FG (%)**	**CSA**
Tenderness	**1,00**	−0,20**	−0,15*	0,12	−0,01	0,18**	−0,19**	−0,06	−0,13*
Total collagen		**1,00**	0,81***	−0,22***	0,05	−0,38***	0,33***	0,17**	0,34***
Insoluble collagen			**1,00**	−0,25***	0,16**	−0,40***	0,31***	0,20**	0,33***
ICDH activity				**1,00**	−0,43***	0,63***	−0,10	−0,57***	−0,21***
LDH activity					**1,00**	−0,36***	0,02	0,35***	0,12
SO (%)						**1,00**	−0,42***	−0,74***	−0,25***
FOG (%)							**1,00**	−0,28***	0,07
FG (%)								**1,00**	0,22***
CSA									**1,00**

Correlation analyses were performed with 495 samples for which all measurements of the indicated variables were available.

*: r > 0.1218 (P < 0.05), **: r > 0.1593 (P < 0.01), ***: r > 0.2018 (P < 0.001).

Tenderness: tenderness score of grilled samples (55-60°C) after 14 days of ageing; Total collagen : total collagen content in mg/g dry matter; Insoluble collagen: insoluble collagen content in mg/g dry matter; ICDH: isocitrate dehydrogenase activity in μmole/min per g muscle; LDH: lactate dehydrogenase activity in μmole/min per g muscle; FG (%): proportion of fast glycolytic muscle fibres; FOG (%):proportion of fast oxydo-glycolytic muscle fibres; SO (%):proportion of slow oxidative muscle fibres; CSA: mean cross-sectional area of muscle fibres in μm^2^.

All these results are in agreement with a great number of other studies where positive correlations between tenderness and collagen solubility (+0.19 < r < +0.24), negative correlations between tenderness and insoluble collagen content (−0.51 < r < −0.42) and negative correlations between tenderness and total collagen content (−0.57 < r < −0.22) were observed by many other authors [3,22,29,31,34,41-43]. However, our result does not fit with the study of Jeremiah & Martin [44] and Silva *et al.*[33] who failed to provide evidence of a significant relationship between intramuscular collagen content or solubility and meat tenderness which was based on shear force values and sensory panel scores. Non significant or inconsistent relationships between connective tissue characteristics and tenderness were reported in several studies [10,32,45-50]. Furthermore, Schönfeldt & Strydom [51] did not find a significant correlation between tenderness and collagen content although there was a weak correlation between tenderness and collagen solubility. This result is in accordance with the conclusion of Mandell *et al.*[52] and McKeith *et al.*[53] who claimed that total collagen content was not a good predictor of overall tenderness of thirteen muscles. One potential reason for these conflicting results is the moderate negative relationship between collagen characteristics and meat tenderness score (r = −0.15 to -0.20, P < 0.05, Table 4) which may be significant only with a large volume of data, and therefore not always detectable in a single experiment. Another potential reason could be differences in cooking temperature across experiments. In the present study, muscle samples were grilled at 55-60°C to assess tenderness which is much lower than in the above studies where the end cooking temperature was generally 70°C, with the exception of some studies such as those of Harris *et al.*[47] and Vestergaard *et al.*[50] where the core temperature was 60-62°C. Cooking has a marked effect on meat toughness due to modification of both the connective and the myofibrillar structures. Findings from Silva *et al.*[33] reported that when meat was cooked at 70°C, the myobrillar component is the main determinant of tenderness [54], particularly in meat from young animals. Although there are conflicting interpretations regarding the relative contribution of the connective tissue and myofibrilar components depending on the heat treatment applied, there is no doubt that differences in collagen content and solubility may be minimised due to denaturation induced by cooking temperatures above 60–65°C [3]. Thus, the greater contribution of collagen components to meat tenderness in the present study may have resulted from the lower cooking temperature.

By combining the data from 43 different experiments, we have shown with significant confidence that a higher level of total collagen as well as more insoluble collagen will lead to a reduction in meat tenderness in meat cooked to around 55-60°C.

### Muscle mean fibre cross-sectional area (CSA)

The mean CSA of muscle fibres are known to vary considerably between muscles [21] and these variations influence beef quality [17]. Muscles in the lowest tenderness class had the highest (3,336 μm^2^) average muscle fibre CSA and conversely muscles in the highest tenderness class had the lowest CSA (2,902 μm^2^) (Table 4). The same result was obtained from the analysis using only the LT muscle (Table 3). On average and across muscles, the correlation between CSA and tenderness score was −0.13 (P < 0.05, Table 3). These results fit well with the negative correlation (−0.11 < r < −0.53) between muscle mean fibre CSA and tenderness found in several studies [3,17,48,55-61].

Opposing these findings, was the study of Seideman *et al.*[62] who found a positive correlation (r = +0.35) between LT muscle fibre CSA and tenderness in steers. Nevertheless, in the same study, this relationship was not evident in bulls. Likewise, Oury *et al.*[26], working on RA muscle from heifers also found no correlation between tenderness and shear force and CSA. However, it should be noted that RA muscle has some specific characteristics in comparison to the LT and TB muscles, especially with respect to the unusual large cross-sectional area of SO fibres and the very low oxidative activity of intermediate fibres (fast oxido-glycolytic) [26].

**Figure 2:**
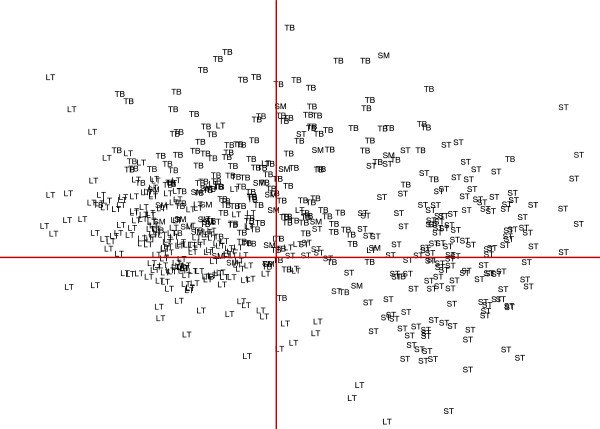
**Plot of the first two principal component score vectors showing variability according to muscle type of the 495 studied muscle samples from different clusters.** ST: *Semitendinosus* muscle; LT : *Longissimus thoracis* muscle; SM: *Semimembranus* muscle; TB: *Triceps brachii* muscle.

In our study, there were mainly LT samples in each cluster (76-94% of all studied muscles), with this muscle previously described as a more tender and oxidative muscle compared to TB, SM, and TB [13,17,61,63]. Several studies [21,26,63,64] demonstrated that LT muscle was characterised by smaller muscle fibre CSA, a trait often associated with a high proportion of oxidative fibres. This was confirmed by the present study, based on the distribution of samples on the plot of the first two principal component score vectors (Figure 2). In fact, CSA was on average 3,215 μm^2^ in LT compared to 3,751 and 4,560 μm^2^ in TB and ST, respectively, (P < 0.01 between the three muscles). In general, glycolytic fibres become larger than oxidative fibres because of the higher requirement for oxygen diffusion within the cells of the latter [8,65].

Moreover, the negative correlation between CSA and tenderness demonstrated for LT muscle was not evident within other muscles presented in this study. The cluster analysis was repeated using only ST and TB muscles (data not shown) with no significant relationship between CSA and tenderness.

According to Crouse *et al.*[59], working on LT muscle from 15 animals, muscle fibre CSA is negatively correlated to sensory tenderness at early periods of post-mortem ageing (1 to 3 days) but not significantly after 14 days. This would suggest that post-mortem proteolysis during storage lowers the negative effects of muscle fibre CSA on tenderness observed soon after slaughter [55,59]. However, our results contradict this suggestion, given that a CSA/tenderness relationship was still evident even though the samples had been aged for 14 days. Thus, our study clearly demonstrates that lower tenderness is associated with a high average muscle fibre CSA, particularly in the LT muscle even after 14 days of ageing.

### Fibre type and mitochondrial enzymes

Some studies [17] considered that metabolic type of fibre may be more directly implicated in tenderness than mean CSA of muscle fibres.

In Table 3, muscles in the highest tenderness group had the highest mitochondrial enzyme activity (ICDH), the highest proportion of SO muscle fibres (25% *vs* 23%) and the lowest proportion of FG muscle fibres (52% *vs* 54%). Correlation analysis across muscles confirmed that the proportion of SO muscle fibres was positively correlated with tenderness score (r = +0.18, P < 0.01) whereas the proportion of FOG muscle fibres was negatively correlated with tenderness score (r = −0.19, P < 0.01, Table 4). These results indicate that SO muscle types favour beef tenderness as observed by Zamora *et al.*[7] and Crouse *et al.*[59] in the LT, by Therkildsen *et al.*[66] in *Longissimus lumborum* and *Supraspinatus* and by Maltin *et al.*[67], Dransfield *et al.*[17] and Jurie *et al.*[63] in a range of different muscles (LT, SM, and TB). However, the relationship between muscle fibre type and beef tenderness has been a subject of debate due to contradictory results generated by numerous experiments carried out in different countries with different animal types and different cuts [8]. These contrasts were also observed in our study, since there was no significant difference in SO and FG muscle fibre proportions between tenderness classes when only the LT muscle was analysed (Table 3).

Some groups working on LT muscle [3,10,68,69] or RA muscle [26], found negative correlations between high oxidative (ICDH activity and proportion of SO fibres) activity, low glycolytic activity (LDH and proportion of FG fibres), and meat tenderness. However, Dransfield *et al.*[17] found that tenderness was negatively correlated to the proportion of FOG fibres rather than FG fibres, which is confirmed by the present study.

Results obtained in this study, including data from all muscle types, confirm the findings of Jurie *et al.*[61] where more tender meat was associated with more oxidative metabolism and smaller (finer) fibre size. In fact, CSA was shown to be positively correlated with the proportion of FG fibres (r = +0.22, P < 0.001) and negatively with the proportion of SO fibres (r = −0.25, P < 0.01, Table 4). Moreover, Renand *et al.*[3], who worked with the LT muscle, found similar results but noted an inverse relationship between oxidative metabolism and tenderness.

Rhee *et al.*[22] found that, across 11 major beef muscles, correlations among all traits were generally the highest in the LT muscle. In fact, in our study, we mainly sampled from LT (76-91%), in the initial statistical analysis. Consequently, results in each cluster are mainly influenced by LT characteristics. However, the relationship between the proportion of muscle fibre types and tenderness were not confirmed with data from the LT muscle only. This indicates that the relationship between muscle fibre types and tenderness, when all muscles are included, is in fact driven by muscles other than the LT.

An additional influencing characteristic on meat quality (the muscle type) demonstrated in this study, LT being the most tender muscle (Figure 2). This is in accordance with several studies [9,10,13,17,43,63,70] in which it was concluded the muscle type played the greatest role in the muscle characteristics and in the determination of meat tenderness. In addition, even greater differences exist between characteristics of connective tissue and of muscle fibre types among the studied muscles namely LT, TB, and ST, as shown in Figure 2.

The relationship between fibre type and tenderness is clearly complex, and it is likely that other variables interact with fibre type characteristics to determine eating quality, in particular, meat tenderness [8]. In addition, there are complex interactions among various biochemical traits across multiple muscles affecting meat tenderness with respect to each individual muscle [22].

## Conclusion

Several muscle characteristics appear to influence beef tenderness, which confirms the complexity of criteria determining meat quality. The large data set of this meta-analysis enables confirmation of well-known negative relationships between tenderness and mechanical properties in one aspect, and between tenderness and collagen characteristics in another aspect. Furthermore, the strength of this meta-analysis with different muscle types lies in its ability to dispel some controversy by showing that oxidative muscle fibre types and a low average muscle fibre cross-sectional area are associated with improved tenderness.

The classes of tenderness studied in this work originated from different muscles sampled from animals of different breeds, sexes, and ages, although we had mainly or only samples from LT muscle in each class of tenderness. Consequently, each cluster of our study may be influenced by LT characteristics. Generally, muscle fibre type played the greatest role in determining tenderness. The volume of data not only brings statistical strength but also a better understanding of the variability according to various criteria e.g. breed, age, and sex, which will be developed in another study. Further work will include more data in the BIF-Beef database in order to identify more variables which may influence tenderness. This biochemical approach needs to be complemented by genomic studies in order to discover new biomarkers, of muscle characteristics, that encode proteins determining muscle traits. A number of other factors such as carcass traits (weight, marbling, ossification, and pH), cooking methods and ageing time, are known to contribute to meat quality, which is why the MSA (Meat Standards Australia) system, which is an integrative approach, was set up. It would be worth integrating muscle traits and genomic markers into this modelling approach.

## Authors’ contributions

SC: data collection, statistical analysis, manuscript preparation; GG and CJ: statistical analysis, manuscript preparation, critical contribution to the final manuscript; BP, DM, and JPB: initial conception of the FiLiCol database and critical contribution to the final manuscript; LJ: data collection for the QUALVIGENE program; JFH: data collection for the GEMQUAL program, design conception, and substantial contribution to the final manuscript. All authors read and approved the final manuscript.
